# Integrating precision cancer medicine into healthcare—policy, practice, and research challenges

**DOI:** 10.1186/s13073-016-0362-4

**Published:** 2016-10-24

**Authors:** Gabrielle Bertier, Jian Carrot-Zhang, Vassilis Ragoussis, Yann Joly

**Affiliations:** 1 Center of Genomics and Policy, McGill University, 740 Dr. Penfield Avenue, Montreal, QC H3A 0G1 Canada; 2 Université Toulouse III Paul Sabatier and Inserm UMR 102, 37 allées Jules Guesde, F-31000, Toulouse, France; 3 Sargent College, Boston University, One Silber Way, Boston, MA 02215 USA

**Keywords:** Cancer, Precision medicine, Genomics, Next-generation sequencing, Health policy, Social-economic challenges

## Abstract

Precision medicine (PM) can be defined as a predictive, preventive, personalized, and participatory healthcare service delivery model. Recent developments in molecular biology and information technology make PM a reality today through the use of massive amounts of genetic, ‘omics’, clinical, environmental, and lifestyle data. With cancer being one of the most prominent public health threats in developed countries, both the research community and governments have been investing significant time, money, and efforts in precision cancer medicine (PCM). Although PCM research is extremely promising, a number of hurdles still remain on the road to an optimal integration of standardized and evidence-based use of PCM in healthcare systems. Indeed, PCM raises a number of technical, organizational, ethical, legal, social, and economic challenges that have to be taken into account in the development of an appropriate health policy framework. Here, we highlight some of the more salient issues regarding the standards needed for integration of PCM into healthcare systems, and we identify fields where more research is needed before policy can be implemented. Key challenges include, but are not limited to, the creation of new standards for the collection, analysis, and sharing of samples and data from cancer patients, and the creation of new clinical trial designs with renewed endpoints. We believe that these issues need to be addressed as a matter of priority by public health policymakers in the coming years for a better integration of PCM into healthcare.

## Background

Precision medicine (PM) is an evolution of contemporary medical practice towards more-efficient prevention and treatment strategies. It can be defined as a predictive, preventive, personalized, and participatory healthcare service delivery model. PM is generally viewed with enthusiasm and optimism by the scientific community [1, 2] and in the media [3, 4]. As cancer is one of the most prominent causes of death and morbidity in developed countries [5], governments have invested massively to make it the ‘poster child’ of PM. Genomic research results are providing us with a more thorough understanding of cancer. It is a complex, multifaceted disease, which challenges established ways to classify, counsel, and treat patients. A number of these results are already actionable, and highly publicized examples demonstrate significant improvements in prevention, survival, and quality of life of patients with certain cancers. Nevertheless, the responsible clinical translation and uptake of precision cancer medicine (PCM) into healthcare systems remains contingent on the proven scientific validity and clinical utility of new genomic sequencing technologies, and on the development of an appropriate policy framework. We define the PCM policy framework as a collection of regulations, laws, guidelines, and policies applying to PCM. This framework will be different in each specific context, whether local, regional, national, or international. Indeed, PCM raises a number of technical, organisational, ethical, legal, social, and economic challenges that have to be taken into consideration in an anticipatory manner to ensure the smooth integration of PCM into the healthcare system.

Here, we discuss key challenges and opportunities regarding the development of a healthcare policy framework for PCM. After describing the current contribution of genomics to PCM, in prevention, classification, and treatment, we highlight a few areas where there is a clear need for new standards. These include genomic data production, analysis, and sharing, as well as clinical trial design and cost assessment of personalized therapies. Finally, we identify a number of fields where more research is needed before policy can be implemented.

## The contribution of genomics to PCM practice

In this section, we highlight how recent genomic research results have already started to impact the practice of medicine, both in cancer prevention and treatment, as summarized in Table 1. Nevertheless, a number of challenges remain, which are also summarized.

**Table 1:** The contribution of genomic information to precision cancer medicine

The contribution of genomic information to precision cancer medicine	Typical example(s)
Cancer risk reduction	Genetic testing of *BRCA1/BRCA2* in hereditary breast cancer and ovarian cancer; *MSH2/MSH6/MLH* in hereditary nonpolyposis colorectal cancer; *RB1* in retinoblastoma
Early detection	Liquid biopsies
Accurate diagnosis	Using molecular markers in tumor classification
Targeted therapy	EGFR inhibitors to treat *EGFR* mutation carriers; BRAF inhibitors to treat *BRAF* V600E carriers; Tyrosine-kinase inhibitor to treat BCR–ABL fusion protein

### Cancer prevention

Genetic testing has already been integrated into routine cancer primary prevention (for example, risk reduction) as well as secondary prevention (for example, screening and early detection) strategies. One clear example is screening in the *BRCA1/BRCA2* genes followed by adapted preventive measures (intensified mammography or removal surgery), which have been shown to significantly reduce breast cancer-associated risks among *BRCA1/2* mutation carriers [6, 7]. More and more risk-prediction models, such as BOADICEA (Breast and Ovarian Analysis of Disease Incidence and Carrier Estimation Algorithm), BRCAPRO, and IBIS (International Breast Cancer Intervention Study), consider genetic status (in *BRCA1/2* and in other common germline variants associated with a small increase in risk [8, 9]) along with other information, including age, ethnicity, family history, lifestyle, and environmental factors, to assess the risk of an individual developing breast cancer [10]. Public health measures have been taken to create stratified prevention and screening programs based on these more precise individual risk calculations [11]. The use of next-generation sequencing (NGS) technologies, which are more rapid and high-throughput than traditional Sanger sequencing, could allow for the genetic testing of mutations in all cancer-susceptibility genes for a large population of individuals, regardless of their family history of cancer [12]. Thanks to the drastic fall in costs of NGS technologies [13], population-based genetic testing using NGS might soon allow for a more systematic identification of cancer-susceptibility mutation carriers at an acceptable cost for the healthcare system.

Another promising avenue to reduce cancer burden is to enable the detection of cancer cells at the earliest possible time. Recently, sizable research efforts have been dedicated to the field of ‘liquid biopsies’—the detection of small amounts of circulating tumor DNA in the blood of patients—before tumors are visible though imaging [14–16]. Although the accuracy of detecting cancer-related events in liquid biopsies requires further improvement, the concept of cancer early detection and treatment outcome prediction from a simple blood test has prompted significant hope for many cancer patients and interest from the private sector; Illumina has recently launched a spinoff company, GRAIL [17], that aims to market a simple blood test for early detection of cancer in asymptomatic patients.

### Tumor classification

The increased use of NGS in research has enabled the development of new strategies to classify tumors according to their mutation status or other biochemical features, rather than their histology or tissue of origin. For instance, a recent study showed that the sequencing of a panel of genes related to brain tumors can be used in routine neuropathology diagnostics and enables the identification of glioma molecular subgroups [18]. Another study identified potential drug targets in 85 % of the samples the authors analyzed (169 of 200) by using gene panel sequencing on samples where the site of the primary tumor was unknown [19]. Some of these molecular findings are being included in new guidelines, such as the recent World Health Organization classification of tumors of the central nervous system [20], which includes a number of molecular markers.

### Treatment

The use of treatment options that specifically target genomic alterations found in tumors has fundamentally changed the field of cancer therapeutics. Indeed, these ‘targeted therapies’ act on specific mutations identified as ‘drivers’ of cancer progression (for example, erlotinib and gefitinib inhibiting tumors with *EGFR* mutations in lung cancer; vemurafenib and dabrafenib inhibiting *BRAF* mutations in melanoma; imatinib and dasatinib targeting *BCR-ABL* translocations in chronic myeloid leukemia; or olaparib inhibiting *BRCA1/BRCA2/PALB2* in ovarian cancer). These have already clearly benefited patients, with improved treatment efficiency and reduced toxicity in non-tumor cells [21] as compared with traditional chemotherapy and radiotherapy. Another research field that is generating significant hope to improve our capacity to treat cancer is that of immune therapy. Indeed, many newly developed targeted therapies based on immune checkpoint-inhibiting processes can induce immune response and rapid tumor regression as a result of a decrease in immunosuppression [22–24]. Combining targeted therapy with immunotherapy is an extremely promising strategy to improve clinical outcomes for cancer patients [25–28].

### Challenges ahead

Still, to date, there are fewer than 30 approved pharmacogenomic drugs for cancer [29], which benefit a relatively low number of patients. Their efficiency has been limited, notably owing to intra-tumor heterogeneity [30, 31] and the development of resistance mechanisms which remain poorly understood [30, 32]. Even though the technologies are becoming more accurate and cheaper by the month, the turnaround time from obtaining genomic material, to accurate diagnosis, and to effective drug prescription still needs to be shortened. A recent project using whole-genome sequencing (WGS) showed that the median time from collecting a patient biopsy to delivering the personalized cancer treatment report was 58 days, still considerably above the 10- to 14-day delay that most patients and physicians would find acceptable [33].

In addition, the impressive advancements [34] and frustrating hurdles associated with the use of genomics in the field of oncology should not allow us forget that, to truly deliver individualized cancer solutions, we will also need to achieve a better understanding of the contribution of patients’ environmental, lifestyle, and psychological factors to cancer development and progression.

## Existing standards challenged, new standards needed

To date, genomic information is collected from a minority of cancer patients, usually in the context of clinical research rather than standard-of-care procedures. However, the situation is evolving rapidly, and the penetration of NGS technologies in the clinical realm has prompted the development of new laboratory guidelines and standards for NGS data production, analysis, and sharing. These efforts have been made by a variety of groups and institutions around the world, which resulted in the publication of numerous partially overlapping guidelines, some extremely general [35] and others focusing on specific diseases, or specific steps in the process, such as the return of results to patients and clinicians [36, 37], or the development of specific bioinformatics pipelines for NGS data analysis [38].

In 2014, Bennett and Farah [31] had already identified more than 15 guidelines applicable to the field of oncology, and more have been published since, such as the European guidelines for clinical NGS [39]. More recently, Nicol and colleagues [40] have eloquently referred to the precision medicine regulatory landscape as a ‘soup’. In addition to the publication of new guidelines, important implementation initiatives have been undertaken in the field of genomics. Two such notable initiatives taking place in the USA are the National Human Genome Research Institute's Implementing Genomics in Practice (IGNITE) project [41], and the National Academies of Sciences, Engineering and Medicine's roundtable on translating genomic-based research for health, which recently published a workshop summary on applying an implementation science approach to genomic medicine [42]. This is of key importance to ensure that guidelines, once published, are actually followed by clinicians and the PCM medical community at large [43–45]. Aside from the clear need for more international and inter-sectorial collaboration in this field to avoid policy redundancy and misalignment, and to ensure efficient implementation, here we highlight significant issues that are specific to PCM. These issues relate to the production, analysis, interpretation, and sharing of cancer patient samples and data, as well as to the need for new clinical trial designs, renewed clinical endpoints, and ethical, legal, and social norms.

### Next-generation sequencing clinical data production

Tumor samples extracted from patients can suffer from low quantity, quality, and purity of tumor cells. Formalin-fixed paraffin-embedded (FFPE) samples are the current gold standard, and most commonly used in clinical laboratories because they can be easily archived and offer a good accuracy for cell morphology-based diagnostics. However, FFPE-derived DNA is usually highly degraded and contaminated by proteins. This low DNA and RNA quality and high DNA fragmentation rate yields a high sequencing error rate. Fresh-frozen samples, by contrast, generally ensure access to the best quantity and quality of tumor DNA, and therefore have certain advantages over FFPE samples in detecting cancer-driving events. However, fresh-frozen samples are currently not widely used in routine clinical molecular analysis, and are not always available for all cancer patients. Tumor DNA can also be extracted from diagnostic biopsies, but this approach is limited because the size of biopsies performed for diagnosis purposes is usually kept to a minimum [46].

Considering the usefulness of obtaining tumor genomic information for cancer diagnosis, prognosis, and treatment, important steps have to be taken to enable the development of renewed standards for state-of-the-art molecular-pathology procedures. This can be achieved by reforming practices of surgeries and pathology laboratories in order to ensure that tissue extraction, preparation, and storage methods are geared towards maximum preservation of DNA (or other molecular features, such as RNA and the methylome) rather than cell morphology, while ensuring maximum safety for patients. However, changing practices and standards in a specialty service takes time, and relies on the commitment of all stakeholders involved in the sample preparation process, from surgeons to pathologists to laboratory technicians. In addition, although guidelines can be issued by professional societies or other government institutions, their implementation has to be adapted for each local laboratory.

After sample collection, steps should be taken at the level of DNA extraction, library preparation, and sequencing experiment design to extract enough high-quality material to perform molecular testing. Significant research efforts have been dedicated to determining how to counteract the effect of low DNA quantity by using a higher sequencing depth. Just how much coverage is enough to ensure maximum sensitivity and specificity of mutation detection is still a matter of debate, and clinical standards should be developed.

### Next-generation sequencing data analysis

Once the sequencing data are collected, and before data interpretation can take place, additional steps have to be taken to estimate tumor purity and to evaluate the amount of contamination by non-tumoral DNA. Bioinformatics pipelines should be adjusted to counter this effect and accurately call somatic variants present in the tumor. Once again, research efforts have been made to establish a computational platform for clinical analysis of sequencing data from FFPE samples [47]. However, formal clinical standards are still needed.

As mentioned above, another hurdle that has to be overcome is tumor heterogeneity. Mutations that are present in subclones within the tumor population, in a low allelic fraction, are difficult to identify [48]. However, their substantial role in generating resistance mechanisms makes them a key element in treatment decisions. Indeed, precision therapies that target and eliminate the major clone also change the tumor environment and provide space for the expansion of minor clones. The accuracy of detecting subclonal mutations partly relies on the computational pipeline used, and several studies have focused on comparing the performances of different variant-calling algorithms [49–51]. However, no rigorous computational tool has been validated for clinical genetic testing in cancer.

There is a clear need to test the performance of bioinformatics software and pipelines for the clinical analysis and clinical interpretation of NGS data and to have them approved by regulatory bodies such as the US Food and Drug Administration (FDA), its sister agency the Centers for Disease Control and Prevention (CDC), or the European Medicines Agency (EMA) [52]. Considering that current practices are highly divergent, as highlighted by the Children’s Leadership Award for the Reliable Interpretation and Appropriate Transmission of Your Genomic Information (CLARITY) challenge [53] and, more recently, by the Clinical Sequencing Exploratory Research (CSER) consortium [54], these institutions have launched initiatives to develop or compare the performance of statistical models and bioinformatics tools fit for NGS data analysis, and the CDC recently published their recommendations [38]. However, those recommendations are focused on detecting germline mutations, which are an order of magnitude less complex and heterogeneous than somatic cancer mutations.

### Data interpretation and sharing

To make clinical sense of the cancer genome of an individual patient, in addition to using specialized bioinformatics tools to predict the functional effect of specific alterations, one has to compare it with thousands of other non-cancer and cancer genomes. A series of filtering steps have to be performed to identify the one or few mutations that are of interest among the thousands of alterations found in a patient’s tumor, each relying on accessing comprehensive and curated databases. In the description below, we are taking the example of the attribution of a targeted treatment, but the same principles are used when looking for a prognosis, treatment side-effect, or resistance biomarker.

When possible, mutations that are common in the general population, and therefore unlikely to have caused the cancer, should be excluded. To perform this step, one has to access large databases of population allele frequencies, such as the 1,000 genomes project [55], dbSNP [56], the Exome Aggregation Consortium (ExAC) [57], and the Exome Variant Server [58]. Although these exist, they are incomplete, biased towards genomes of European ancestry, and not well curated [59–61]. Furthermore, among the multiple potentially causal somatic mutations found in a tumor, one has to identify those that are driving oncogenesis, as opposed to ‘passenger mutations’ which have no effect on cancer development. This is usually attempted by searching for mutations already found to drive tumors in other cancer patients. These searches are performed in large-scale publically accessible databases such as the Catalogue of Somatic Mutations in Cancer (COSMIC) [62], the cBioPortal for Cancer Genomics [63], the Therapeutically Applicable Research to Generate Effective Treatments (TARGET) [64], the Pediatric Cancer Genome Project [65], or the ‘My Cancer Genome’ tool [66]. In addition, most clinical research laboratories use their own patient genome databases. Those can be difficult to share with the wider community if they contain specific patient information or if they were obtained in a strict clinical setting with no consent to share the data for research. This phenomenon has been particularly reported in the field of rare diseases [67], and the recent European Society of Human Genetics guideline specifically encourages clinical laboratories to share these locally accumulated data [39]. Researchers can also apply for access to raw sequencing data created by two large-scale international cancer sequencing initiatives, namely The Cancer Genome Atlas (TCGA) [68] and the International Cancer Genome Consortium (ICGC) [69]. Nevertheless, those databases would be more valuable if they would systematically gather clinical and demographic data. The next evolution of the ICGC project, ICGCmed, promises to collect a much richer dataset to enable personalized medicine [70]. Similarly, the Sharing Clinical Reports Project (SCRP) has been initiated by ClinVar and aims to collect the identification and clinical interpretation of *BRCA1/2* variants. The Global Alliance for Genomics and Health (GA4GH) [71] also launched the BRCA challenge demonstration project [72], providing an efficient platform with all *BRCA* mutations collected from patients around the world, together with their phenotypic characteristics. Following this push from the research community for a more systematic sharing of all patient cancer genome data, the for-profit diagnostic company Ambry Genetics recently announced the publication of more than 10,000 whole exomes from cancer-diagnosed clients in the open access database AmbryShare [73, 74]. To increase the pool of cancer exome data, and to push for a faster implementation of PCM, the company Strata Oncology has raised capital to offer free DNA sequencing to 100,000 US cancer patients [75].

Once likely driver mutation(s) have been identified in the patient tumor genome, the next step is to find those that are ‘actionable’, or targetable by a therapeutic agent. If such an agent exists, and is manufactured, the decision on whether and how to use it to treat the cancer patient depends on a number of factors. In the best-case scenario, the agent exists and has been approved by the local regulation agency for patients with the same characteristic (such as cancer type and stage, age, previous treatment course). Accessing approved drug databases is relatively straightforward, notably with the use of pharmGKB [76], an online resource that provides a list of most, if not all, pharmacogenomics agents approved or under consideration by the FDA, EMA, and others, and containing a wealth of information on each agent. In a more likely scenario, the agent might have been approved to treat patients with a different cancer or age group (as often happens for pediatric patients, as significantly more trials are launched with adults as compared with children). In this case, the treating oncologist has to decide whether to provide the drug ‘off label’ to the patient or to add the patient to an existing clinical trial where he/she could also receive the drug and be monitored (providing funding is available). Oncologists make these decisions based on a set of criteria that can include existing formal or informal hospital policies, their knowledge of the clinical features of the patient, and the practice of their colleagues in the field. Once again, accessing the information about existing clinical trials requires the use of up-to-date and reliable databases. The US National Institutes of Health (NIH) maintains a relatively complete database freely accessible online [77], which includes trials registered in the USA and around the world. Most clinicians rely on their professional networks to have access to information on existing trials, whether it is directly with agent manufacturers, pharmaceutical companies, or other clinicians. Another issue is that of accessing results of clinical trials, and sharing information on the positive or adverse reactions of patients to treatment regimens. There again, efficient and systematized reporting strategies have to be put in place so that clinicians and patients can take informed decisions on the course of care [78].

Finally, data sharing is necessary to enable a more sustainable and reliable discovery and exploitation of biological links between compounds and diseases [52]. The pharmaceutical industry is getting increasingly involved in this research. AstraZeneca, for example, announced early in 2016 that it would analyze the genomes of two million patients to help inform its drug discovery research and share the insights generated by the sequencing, including information on variant data and drug targets, with the research community [79].

### Test selection

Although most cancer patients who undergo genetic analysis today have access to targeted tests, current NGS strategies include sequencing of a panel of known cancer genes, or the entire protein coding region of the genome (whole-exome sequencing, WES), WGS or sequencing the entire transcriptome (RNA-seq). Ordering the right test for a particular cancer patient requires a comprehensive evaluation of their clinical features, at an acceptable turnaround time and affordable cost [80]. Gene panel sequencing is the cheapest and perhaps the most effective method to identify an ‘actionable’ mutation because, to date, only a limited number of cancer genes can be matched to a targeted therapy. The WES and WGS strategies are the most ‘hypothesis-free’ approaches, and they are more likely to uncover novel mutations that can be matched to a new drug that has not been used in cancer before. However, WES and WGS are more costly in data production, storage, and in computation. In addition, owing to the biased coverage of these techniques towards low-complexity regions in the genome, validation assays have to be performed for each of the candidate variants uncovered through WES or WGS. This entails non-negligible additional time and costs [81, 82]. WGS has several advantages over WES, including the identification of regulatory variants that affect gene expression, and structural variants in which the breakpoints are located outside exons. RNA-seq is complementary to DNA sequencing as it directly measures gene expression, splicing variants, and gene fusions. In the future, the tumor and matched normal whole-genome of each patient could be sequenced at diagnosis and be made available in their medical records before clinicians make treatment decisions. However, before that time comes, new guidelines are needed to help clinicians decide which is the most appropriate test to prescribe to their patients.

### Cost of drugs and clinical trials design

Calculating the absolute cost-effectiveness of personalised interventions is far from easy. In a systematic review published in 2014, the authors failed to determine whether ‘individualized medicine interventions’ were more or less cost effective than standard-of-care interventions—their admittedly disappointing answer to this question was “it depends” [83].

PCM could be less cost efficient for a number of reasons: because the cost of developing and marketing a biological drug is extremely expensive, this investment is justified only if the target population for the drug is large, and if the benefits and gains in ‘quality-adjusted life years’ (QALY) are sizable. By definition, targeted therapies have a small beneficiary population size, and are therefore less cost-effective. One should also consider the potential need for many more clinical trials than for untargeted chemotherapies [84]. In addition, personalized therapies are some of the most expensive drugs currently on the market [85, 86]. However, a number of elements complicate this simplistic picture, leading many to argue that traditional cost-effectiveness calculations have to be re-thought in the context of PCM [87]. First, improved ‘personalised’ risk prediction models could allow substantial healthcare savings by improving chances for early detection and management of cancer [12]. Second, although niche oncology drugs are expensive, the costs of biomarker detection are diminishing (currently between $100 and $5000 per patient) [87, 88], notably owing to the progress made in the fields of bioengineering and the increased use of nanotechnologies [89]. Third, contrary to personalized drug development, the use of genomic tests to adjust the dosage of an already approved medication or to substitute an approved drug for another seem to be intuitively very cost efficient. Doing so allows for a more rational and safe drug use (i.e., less hospitalization from adverse effects) at little extra cost [90]. Indeed, many cancer patients harbor mutation patterns that are already targeted by approved agents, either in other cancers or other diseases. This significantly expands the possibilities for drug repositioning, removing the cost of phase I and phase II experiments, and moving directly to phase III trials or even N-of-1 trials [78]. These smaller, more targeted clinical trials with more strictly selected patients, and with a higher chance of success, could be highly cost effective [90, 91]. Fourth, the potential market size of PCM drugs might not be that small. Indeed, research results show that a limited number of pathways are implicated in tumorigenesis, and regulate mechanisms comprising the “hallmarks of cancer” [92]. Patients with pathophysiologically different cancers sometimes share the same mutation patterns, and could therefore be included in the same trials. New types of dynamic multi-armed trials based on the patients’ molecular patterns are already under way [93–97]. Admittedly, their actual cost efficiency and success rate remain to be determined. Nevertheless, all these factors combined have led some authors to predict that the market might be flooded by a ‘tsunami’ of targeted therapies in the near future [98].

Finally, it has been suggested that a renewed, more comprehensive approach has to be taken when defining intervention endpoints. Gold standards such as QALYs, progression-free survival, and clinical utility have to be complemented by other ‘real-world’ measures taking into account actual patient and clinician experiences with the treatment, as well as more general societal preferences and values [99]. In addition, careful evaluation of psychosocial as well as economic costs of adverse effects to therapies have to be included in cost-efficiency calculations [90]. These renewed endpoints, combined with dynamic reimbursement models, such as reimbursement with evidence collection [42], could promote an efficient and timely integration of PCM into the public healthcare system.

### Challenging the traditional legal and ethical norms

Without necessarily creating a complete new set of legal and ethical issues, the advent of PM has made the border between research and healthcare increasingly porous [100–102]. This uncertainty challenges policymakers to find new policy tools and solutions to protect traditional principles and norms such as informed consent, return of results, privacy and confidentiality, and benefit sharing [103–105]. Given that research is an increasingly international endeavor, whereas healthcare is still defined at the national or regional level, these questions will need to be answered at different geographical layers, while promoting normative coherence and integration. Professional organizations, industry, and non-governmental organisations—such as the Public Population Project in Genomics and Society (P3G) [106] and GA4GH—are all contributing to this normative process, and they would be well advised to collaborate closely to facilitate policy interoperability and avoid the concurrent development of misaligned norms.

## Research needed before implementation

### Identification of unresolved scientific questions

As argued by Blay and colleagues in a recent review [84], the field of PCM would benefit from the systematic, periodic, and, if possible, consensual establishment of research priorities by the scientific community. This would be extremely helpful for policymakers to target promising fields of research and invest in the necessary improvements of care. In addition, it would contribute to the design of governance structures that anticipate future societal needs, and guide the development of technologies such as genomics towards resolving current and future issues [107]. Such publications could stem from high-level international meetings such as the recent International Congress on Personalised Healthcare, which took place in Montreal in June 2016 [108].

### Non-genetic aspects of cancer

In making decisions on how to implement PCM, policymakers also have to take into account elements that greatly influence cancer incidence and prevalence in the population, but which are not genetic. Several types of environmental and behavioral risk factors also have to be considered: first, those that have been unequivocally associated with an increased cancer risk, such as smoking, radiation, sun exposure, and certain infections (for example, the human papillomavirus causing cervical cancer); and second, those that might have a protective effect on cancer such as a healthy diet and regular physical activities. For those two types of factors, clear policy guidance and prevention campaigns targeting the general population can be designed. By contrast, ensuring public buy-in to prevention through changes in lifestyle remain very challenging for public health policymakers [109–113]. Finally, certain factors have been shown to have an equivocal effect on cancer risk, progression, and survival (for example, the use of oral contraceptives, which has both protective and risk increasing effects on breast and ovarian cancer [114, 115]). There are also important social determinants of cancer incidence and mortality [116, 117], which have a tendency to be overshadowed by the appeal of new technologies in genetics and the media portrayal of the promises of PM. Clearly, more research and high-quality evidence are needed to better understand the joint impact of genetic and non-genetic factors on cancer risk and survival [118]. Advancement in technology that generates ‘hype’ [119] should not overtake research funding, and policy efforts to reduce inequalities and incidence of deprivation-associated cancers, such as lung and head and neck cancer [120], should be maintained.

### General questions on precision medicine

In addition to the issues described, which can be specific to cancer, a number of issues pertain more generally to the implementation of PM.

#### Patients’ preferences: new evidence needed

Published evidence on the acceptance of genomic sequencing by patients is compelling. Patients are overwhelmingly positive towards these new techniques [90, 121–123]. Nevertheless, more comprehensive research is needed to understand patient preferences in a variety of cancer contexts. From existing evidence, it is clear that, if PM intervention is offered in the context of a clinical research project, as a ‘last chance’ for patients who do not respond to standard-of-care treatments, they are highly likely to agree to participate in risky trials and to test new therapies. Could the same conclusion be drawn in cases where chemotherapy is associated with a better or a similar prognosis compared with that of a PCM intervention? In addition to patients, it is important to consider the opinions of healthcare professionals and the general public on PCM [84]. Real-world data are greatly needed to understand patients’ experiences with different toxicities, their individual tolerance to risk and risk–benefit balance, and what they consider a most desirable outcome from a treatment [90].

#### Access to precision medicine

Today, access to PM is restricted to certain large-scale cancer centers in developed countries, and is not available in smaller centers in most of the developing world. Access can also be dependent on regional infrastructure, market size, and the political capacity to negotiate special agreements with pharmaceutical companies for access to new treatments. At the national level, access to certain off-label or clinical trial treatments can still be dependent on the hospital where the patient receives care, and on the presence of clinical researchers among the oncology team. Straightforward solutions still have to be put forward to ensure an equitable access to PCM, both in clinical research and when it becomes standard of care, nationally and internationally.

#### Infrastructures and education

Undoubtedly, significant investments have to be made in two areas to enable PM. First, infrastructures have to be put in place to produce, store, link, and share the data. This includes sequencing technology equipment, secure high-throughput computing infrastructures, as well as reliable and standardized electronic health record systems integrating genomic and phenotypic patient information. Second, the general public, clinicians, and other stakeholders such as insurance providers all have to be educated about PM. Informing the general public will increase the level of participation in crucial initiatives such as large-scale population sequencing, as well as disease-specific research. Clinicians at all levels also need to be educated and engaged in PM initiatives, and a greater number of genetic counsellors integrated within healthcare teams. This is crucial to ensure an efficient uptake of available techniques and treatment options, so that PM research efforts are actually translated to daily patient care [104, 124]. These education efforts have to be made on top of the publication of official clinical practice guidelines [90, 125].

#### PM implementation: a local question?

Solutions need to be designed to reconcile the international collection of scientific evidence of clinical validity and clinical utility of PM interventions, and the necessary local calculations of cost-effectiveness and reimbursement procedures. Indeed, these last considerations are highly dependent on the healthcare system organisation, guiding principles, political, cultural, and economic context. Individual nations alone will be able to make implementation choices that they deem appropriate in their local context. Nevertheless, a number of large-scale international initiatives have taken place that aim to share implementation results and benefits from PM. These are listed and described in a recent review by Manolio and colleagues [126]. Despite this, as concluded by the authors, more globalized efforts are needed to make sure as little unnecessary duplication of efforts as possible is performed.

## Conclusions

We have attempted to highlight some of the salient issues in the implementation of PCM. As summarized in Table 2, we have described a number of areas in which new standards need to be established before PCM implementation, including the collection, analysis, and sharing of cancer patient samples and data, as well as the need for new clinical trial designs. In addition, we have identified areas where significant research efforts are needed before PCM policies can be established, notably regarding the non-genetic aspects of cancer. This discussion is, by choice, more selective than comprehensive, and some elements might be missing from this list. However, we have identified broad themes that we think should be tackled by policymakers who are presently taking decisions on PCM implementation. A better understanding of the complex scientific and policy issues posed by PCM by all stakeholders is desirable in order to find solutions and improve the translation of PM in public and private health systems.

**Table 2:** Integrating precision cancer medicine into healthcare—key challenges and opportunities

Area	Challenge	Opportunity
Medical Practice		
Detection	Many cancers diagnosed too late	Liquid biopsies
Turnaround time	Time from sample collection to clinically actionable result often too long	Optimization of sample collection and data analysis pipelines
Treatment	Limited efficiency of targeted treatments	Research on resistance mechanisms and tumor heterogeneity, and use of combined targeted and immune therapy
New standards needed		
Publication and implementation of clinical guidelines	Multiple partly overlapping guidelines published, poor international and inter-sectorial operability	Collaborations between agencies such as the FDA, the EMA, Health Canada, and the NHS. Implementation projects (IGNITE and others)
Sample collection	Current gold standard (FFPE) suboptimal for genomic data analysis.	Standardization and implementation of new cancer sample collection strategies (for example, fresh frozen) to maximize quality, quantity, and purity of tumor cells.
Sample preparation and analysis	Suboptimal DNA extraction, library preparation, and sequencing protocols for molecular testing of cancer samples	Implementation of new standards to counteract unavoidable cancer sample limitations (low quality, quantity and purity, high heterogeneity)
Cancer genomic data analysis	Current bioinformatics pipelines and software suboptimal for the identification of actionable cancer mutations	Development and clinical validation of bioinformatics tools and pipelines for a thorough molecular analysis of tumor samples (including main and subclonal mutations, and cellular context)
Cancer genomic data sharing	Genetic diversity of the general population and cancer patients poorly represented in current publically available databases.	Development of improved and curated cancer-specific and population databases
	Widely variable data sharing policies among clinical institutions and research projects	Alignment of international policies on cancer patients’ data sharing
	Clinical trials and compound registration fragmented and patchy	Improve databases of approved compounds and international clinical trial registries
Test selection	Widely variable genetic testing practices for similar cancer patients across clinical institutions	Production of clinical guidelines on genetic test selection (single gene/gene panel/whole exome/whole-genome sequencing)
Clinical trials and cost of drugs	Classical clinical trial designs (large and diverse patient populations) inappropriate to test targeted therapies	New clinical trial designs: drug repositioning tests, ‘n-of-one’ trials, rotation therapies
	Cost-effectiveness of targeted therapies widely contested	Thorough examination of cost-effectiveness of cancer genomic medicines, taking into account new clinical trial designs
Intervention endpoints	Traditional endpoints and measures (QALYs) ill-adapted to personalized medicine interventions	Renewed, more holistic intervention endpoints, including patient experience, societal preferences, and values
Policy, ethical and legal norms	Border between research and healthcare increasingly porous	Development of adapted, international and interoperable ethical and legal norms (GA4GH, P3G)
	Higher uncertainty associated with the clinical significance of genomic information	
	Tension between international research endeavors and national healthcare systems	
Pre-implementation research needed		
Identify priorities	Need for a systematic identification of unresolved scientific questions	International conferences and expert reviews in PCM
Non-genetic aspects of cancer	A number of elements still poorly understood • Environmental factors • Behavioral factors • Social determinants of cancer incidence and survival	Support for targeted research in those domains, while continuing efforts to reduce known factors leading to increased cancer incidence and prevalence (smoking, alcohol consumption, and social deprivation)

*EMA* European Medicines Agency, *FDA* Food and Drug Administration, *FFPE* formalin-fixed paraffin-embedded, *GA4GH* The Global Alliance for Genomics and Health, *IGNITE* Implementing Genomics in Practice, *NHS* National Health Service, *PCM* precision cancer medicine, *P3G* Public Population Project in Genomics and Society, *QALY* quality-adjusted life years

Potential future avenues include promoting investments in infrastructures and promising research fields, an increased effort in education and training of healthcare professionals about PCM, and the design of more suitable models to evaluate the cost-effectiveness of personalized interventions. We are extremely positive on the potential of PM to improve cancer care in the future; however, in the current transition period, it is of prime importance that the public be accurately informed and engaged on this topic. Most cancer diagnostics today still come with a grim prognostic, and it would be unfortunate to see patients develop inappropriate expectations of ‘miracle’ PCM cures. We therefore call for the promotion of a balanced portrayal of what PCM is, its limits, and what it can do for cancer patients today, both in the research literature [127–129] and in the media.

## Abbreviations

BOADICEA: Breast and Ovarian Analysis of Disease Incidence and Carrier Estimation Algorithm
CDC: Center for Disease Control and Prevention
CLARITY: Children’s Leadership Award for the Reliable Interpretation and Appropriate Transmission of Your Genomic Information
COSMIC: Catalogue of Somatic Mutations in Cancer
CSER: Clinical Sequencing Exploratory Research
EMA: European Medicines Agency
ExAc: Exome Aggregation Consortium
FDA: Food and Drug Administration
FFPE: Formalin-fixed paraffin-embedded
GA4GH: The Global Alliance for Genomics and Health
IBIS: International Breast Cancer Intervention Study
ICGC: The International Cancer Genome Consortium
IGNITE: Implementing Genomics in Practice
NGS: Next-generation sequencing
P3G: Public Population Project in Genomics and Society
PCM: Precision cancer medicine
PM: Precision medicine
QALY: Quality-adjusted life years
SCRP: Sharing Clinical Reports Project
TARGET: Therapeutically Applicable Research to Generate Effective Treatments
TCGA: The Cancer Genome Atlas
WES: Whole-exome sequencing
WGS: Whole-genome sequencing
